# Optimization of Astilbin Extraction from the Rhizome of *Smilax glabra*, and Evaluation of Its Anti-Inflammatory Effect and Probable Underlying Mechanism in Lipopolysaccharide-Induced RAW264.7 Macrophages

**DOI:** 10.3390/molecules20010625

**Published:** 2015-01-06

**Authors:** Chuan-Li Lu, Yan-Fang Zhu, Meng-Mei Hu, Dong-Mei Wang, Xiao-Jie Xu, Chuan-Jian Lu, Wei Zhu

**Affiliations:** 1 Guangdong Provincial Academy of Chinese Medical Sciences, Guangzhou 510006, China; E-Mails: luyixiao-0707@163.com (C.-L.L.); iris287@126.com (D.-M.W.); xiaojxu@pku.edu.cn (X.-J.X.); luchuanjian888@vip.sina.com (C.-J.L.); 2 School of Fundamental Medical Science, Guangzhou University of Chinese Medicine, Guangzhou 510120, China; E-Mails: yf_zhu01124@126.com (Y.-F.Z.); mengmeihu@126.com (M.-M.H.); 3 Post Doctoral Research Station, Guangzhou University of Chinese Medicine, Guangzhou 510120, China

**Keywords:** astilbin, response surface methodology, *smilax glabra* Rox, anti-inflammatory, inflammatory cytokine, RAW264.7 cell

## Abstract

Astilbin, a dihydroflavonol derivative found in many food and medicine plants, exhibited multiple pharmacological functions. In the present study, the ethanol extraction of astilbin from the rhizome of *smilax glabra* Roxb was optimized by response surface methodology (RSM) using Box-Behnken design. Results indicated that the obtained experimental data was well fitted to a second-order polynomial equation by using multiple regression analysis, and the optimal extraction conditions were identified as an extraction time of 40 min, ethanol concentration of 60%, temperature of 73.63 °C, and liquid-solid ratio of 29.89 mL/g for the highest predicted yield of astilbin (15.05 mg/g), which was confirmed through validation experiments. In addition, the anti-inflammatory efficiency of astilbin was evaluated in lipopolysaccharide (LPS)-induced RAW 264.7 cells. Results showed that astilbin, at non-cytotoxicity concentrations, significantly suppressed the production of nitric oxide (NO) and tumor necrosis factor-α (TNF-α), as well as the mRNA expression of inducible nitric oxide synthase (iNOS) and TNF-α in LPS-induced RAW 264.7 cells, but did not affect interleukin-6 (IL-6) release or its mRNA expression. These effects may be related to its up-regulation of the phosphorylation of p65, extracellular signal-regulated kinases 1/2 (ERK1/2) and *c*-Jun N-terminal kinase (JNK).

## 1. Introduction

Inflammation is an important component of innate immunity and the host response to pathogens [1]. However, uncontrolled excessive inflammation may be detrimental, and contribute to the pathogenesis of many diseases [2]. Pro-inflammatory cells, mainly activated macrophages, mediate most of the cellular and molecular pathophysiology of inflammation by producing cytokines and other pro-inflammatory molecules, including prostaglandins, enzymes and free radicals [3]. LPS is a compound derived from Gram negative bacterial cell walls, which has been proposed as a potent inducer of inflammatory cytokines. In response to LPS, the RAW264.7 cells become potent secretary cells that release various pro-inflammatory mediators, including IL-1β, IL-6, TNF-α, and NO [4]. Thus, LPS-activated RAW264.7 macrophages have been broadly used for evaluating the anti-inflammatory activity of natural products.

It is well known that there is a complex regulatory loop which amplifies and perpetuates inflammatory response. Nuclear factor-kappa B (NF-κB) and mitogen-activated protein kinase (MAPK) signaling pathways are key regulators in this amplifying loop. NF-κB is an important transcription factor complex that regulates the expression of many genes involved in immune and inflammatory response. After being activated, the NF-κB rapidly translocates to the nucleus and activates the transcription of target genes, which encode the pro-inflammatory cytokines, chemokines, and inducible enzymes [2]. MAPK signal transduction pathways are classified into at least three components: p38, ERK1/2 and JNK, which play critical roles in the regulation of cell growth and differentiation as well as in the control of cell responses to cytokines and stressors. Moreover, they are also known to be important for the activation of NF-κB [1,5]. Because of their pivotal role in the amplifying loop of the inflammatory response, NF-κB and MAPKs has become logical targets for new types of anti-inflammatory treatment.

Astilbin (2*R*, 3*R*)-taxifolin-3-*β*-*O*-rhamnoside) was firstly isolated from the rhizome of *astilbe thunbergill* by Hayashi and Ouchi [6], and is also found in many other plants, such as *Psychotria prumfolia*, *Senna obtusifolia*, *Dimorphandra mollis*, *Tithonia diversifolia* [7], *Heritiera littoralis* [8], *Engelhardtia roxburghiana* [9,10], *Smilacis Glabrae* [11], *Smilacis Chinae* [12], *Drimys brasiliensis* [13], *Hymenaea courbaril* L. [14], *Hymenaea stigonocarpa* [15], *Pieris japonica* [16], *etc.* Previous researches indicated that astilbin possesses a potential utilization in both health food and medicine, because of its multiple bioactivities, such as improving immunological liver injury [17], as well as its antioxidant [18,19], anti-inflammatory [9,18,19,20], anti-arthritic [21], and anti-diabetic nephropathy [22] properties.

However, to the best of our knowledge, the anti-inflammatory activity has not yet been fully revealed. Furthermore, there has been no report on its preparation from natural resources, especially from *S. glabra*. In present study, the anti-inflammatory efficacy of astilbin was investigated using LPS-induced RAW264.7 cells. In addition, the ethanol extraction of astilbin from the rhizomes of *S. glabra* was optimized by RSM.

## 2. Results and Discussion

### 2.1. Box-Behnken Design Analysis

In order to study the combined effect of independent variables on the extraction yield of astilbin (EYA), 29 experiments were carried out for different combinations of the extraction parameters using Box-Benhken design, and the results are shown in Table 1. Five runs of these experiments (8, 9, 23, 25 and 26), in which all the factors were set at central levels, were used for evaluating the stability of the experiment, and the remaining 24 runs were for analysis. Model adequacy checking was carried out on the obtained data to determine whether the approximating model would give poor or misleading results [23].

**Table 1:** Uncoded and coded Box-Behnken design with observed and predicted data.

Run	Extract Time (A, min)	Ethanol Concentration (B, %)	Temperature (C, °C)	Liquid-Solid Ratio (D, mL/g)	EYA (mg/g)	
					Observed	Predicted
1	40(1)	55(0)	65(0)	10(−1)	12.25	12.13
2	40(1)	55(0)	65(0)	30(1)	14.35	14.26
3	40(1)	60(1)	65(0)	20(0)	13.91	14.00
4	40(1)	55(0)	75(1)	20(0)	14.40	14.22
5	10(−1)	55(0)	55(−1)	20(0)	13.60	13.75
6	25(0)	55(0)	55(−1)	10(−1)	12.04	12.06
7	10(−1)	55(0)	65(0)	30(1)	14.06	13.99
8	25(0)	55(0)	65(0)	20(0)	14.17	14.39
9	25(0)	55(0)	65(0)	20(0)	14.50	14.39
10	25(0)	55(0)	75(1)	10(−1)	12.80	12.81
11	25(0)	50(−1)	65(0)	10(−1)	12.61	12.73
12	25(0)	55(0)	75(1)	30(1)	14.20	14.40
13	25(0)	55(0)	55(−1)	30(1)	14.05	14.27
14	40(1)	50(−1)	65(0)	20(0)	12.52	12.66
15	10(−1)	50(−1)	65(0)	20(0)	14.04	14.17
16	40(1)	55(0)	55(−1)	20(0)	12.87	13.03
17	25(0)	50(−1)	55(−1)	20(0)	13.78	13.46
18	25(0)	60(1)	65(0)	10(−1)	11.47	11.54
19	25(0)	50(−1)	65(0)	30(1)	13.76	13.66
20	10(−1)	55(1)	65(0)	20(0)	12.32	12.41
21	25(0)	50(−1)	75(1)	20(0)	13.76	13.79
22	25(0)	60(1)	55(−1)	20(0)	13.37	13.14
23	25(0)	55(0)	65(0)	20(0)	14.64	14.39
24	25(0)	60(1)	65(0)	30(1)	14.57	14.42
25	25(0)	55(0)	65(0)	20(0)	14.31	14.39
26	25(0)	55(0)	65(0)	20(0)	14.33	14.39
27	10(−1)	55(0)	75(1)	20(0)	13.62	13.43
28	25(0)	60(1)	75(1)	20(0)	13.55	13.68
29	10(−1)	55(0)	65(0)	10(−1)	12.43	12.32

In general, there are four kinds of relationships between the independent variables and the response variable, linear, interactive, quadratic and cubic. In present study, three different tests, namely the sequential model sum of squares, lack of fit tests and model summary statistics, were performed to evaluate which model would be more suitable for the relationship between the process independent variables (extraction time, ethanol concentration, temperature, and solid-liquid ratio) and the extraction yield of astilbin, and the results are exhibited in Table 2. Quadratic model was found to be the most suitable model for the extraction of astilbin from the rhizomes of *S. glabra*, as its sequential model sum of squares was significant (*p* < 0.0001), but lack of fit tests were insignificant (*p* = 0.3510, >0.05). Furthermore, it had maximum *R*^2^, adjusted *R*^2^, and predicted *R*^2^. By applying multiple regression analysis on the experimental data, the response variable (EYA) and the test variable were related by the following second-order polynomial equation:

EYA = 14.39 + 0.019 A − 0.11 B + 0.22 C + 0.95 D + 0.78 AB + 0.38 AC + 0.12 AD + 0.05 BC + 0.49 BD − 0.15 CD − 0.5 A^2^ − 0.58 B^2^ − 0.29C^2^ − 0.72 D^2^ (1)

**Table 2:** Sequential model fitting for the extraction yield of astilbin.

Source	Sum of Squares	df	Mean Square	*F*-Value	*p*-Value (prob>)	Remarks
Sequntial model sum of squares						
Mean	5306.33	1	5306.33			
Linear	11.52	4	2.88	6.89	0.0008	
2FI	4.10	6	0.68	2.07	0.1086	
Quadratic	5.29	4	1.32	28.19	<0.0001	Suggested
Cubic	0.35	8	0.043	0.84	0.6032	Aliased
Residual	0.31	6	0.052			
Total	5327.90	29	183.72			
Lack of fit tests						
Linear	9.91	20	0.50	14.90	0.0089	
2FI	5.81	14	0.42	12.48	0.0128	
Quadratic	0.52	10	0.052	1.57	0.3510	Suggested
Cubic	0.18	2	0.089	2.66	0.1839	Aliased
Pure error	0.13	4	0.033			
Model summary statistics						
Source	Std. Dev.	R^2^	Adjusted R^2^	Predicted R^2^	Press	Remarks
Linear	0.65	0.5344	0.4568	0.3588	13.83	
2FI	0.57	0.7244	0.5712	0.4870	11.06	
Quadratic	0.22	0.9696	0.9391	0.8505	3.22	Suggested
Cubic	0.23	0.9856	0.9329	-0.1926	25.72	Aliased

Analysis of variance (ANOVA) was performed to investigate the adequacy of the suggested model and identify the significant factors [24], and the results are shown in Table 3. The model *F*-value of 31.85 indicated that the model was significant at *p* < 0.0001. The lack of fit *F*-value of 1.75 and the associated *p*-value of 0.3510 were insignificant due to relative pure error. These suggested that the model terms were significant and applicable. From the corresponding *p*-value of each model term, it could be concluded that two linear coefficients (C and D), three interactive coefficients (AB, AC and BD) and four quadratic coefficients (A^2^, B^2^, C^2^ and D^2^) were significant, indicating the pattern of the interactions between the variables.

**Table 3:** ANOVA for Response Surface Quadratic Model.

Source	Sum of Squares	df	Mean Square	*F*-Value	*p*-Value (prob>)
Model	20.91	14	1.49	31.85	<0.0001
A	4.408 × 10^−3^	1	4.408 × 10^−3^	0.094	0.7637
B	0.14	1	0.14	2.91	0.1100
C	0.57	1	0.57	12.20	0.0036
D	10.81	1	10.81	230.54	<0.0001
AB	2.42	1	2.42	51.56	<0.0001
AC	0.57	1	0.57	12.16	0.0036
AD	0.055	1	0.055	1.18	0.2962
BC	0.010	1	0.010	0.21	0.6513
BD	0.95	1	0.95	20.27	0.0005
CD	0.093	1	0.093	1.98	0.1808
A2	1.59	1	1.59	34.01	<0.0001
B2	2.22	1	2.22	47.27	<0.0001
C2	0.53	1	0.53	11.40	0.0045
D2	3.35	1	3.35	71.37	<0.0001
Residual	0.66	14	0.047		
Lack of fit	0.52	10	0.052	1.57	0.3510
Pure error	0.13	4	0.033		
Cor toal	21.56	28			

### 2.2. Optimization of Extraction Conditions of Astilbin

**Figure 1:**
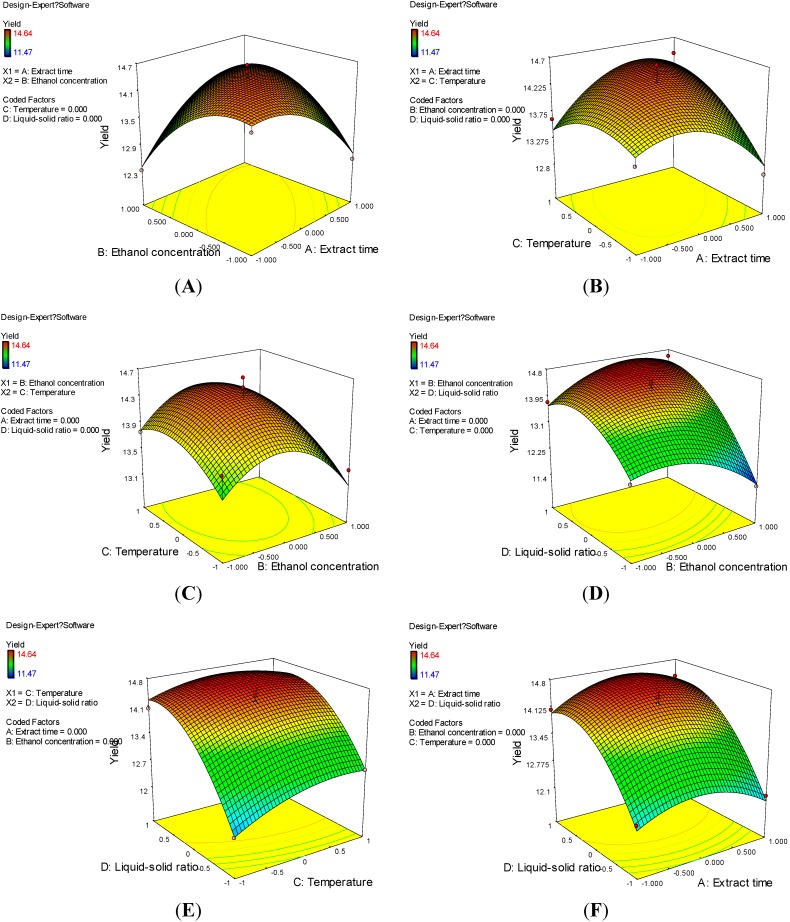
Response surface (3-dimensional) showing the effects of process conditions on the extraction yield of astilbin. (**A**) ethanol concentration-extraction time; (**B**) temperature-extraction time; (**C**) temperature-ethanol concentration; (**D**) liquid-solid ratio-ethanol concentration; (**E**) liquid-solid ratio-temperature; (**F**) liquid-solid ratio-extraction time.

The response surfaces, the graphical representations of the regression Equation-(2), were constructed using Design-Expert 7.0 software. Figure 1 represents a three-dimensional plot demonstrating the relationship between independent and dependent variables. Figure 1A indicated that there is an obvious interaction between extraction time and ethanol concentration while extraction temperature and liquid-solid ratio were kept at the center level. When the ethanol concentration was lower than 55%, the optimal extraction time for the highest EYA ranged from 25–40 min. However, when the ethanol concentration was higher than 55%, the optimal extraction time ranged from 15–40 min. Figure 1B shows the effect of extraction time and temperature on the EYA at the center level of ethanol concentration and liquid-solid ratio. It can be seen that with the temperature and extraction time rising at the beginning, the EYA increased. However, when they exceeded a certain value, the value of EYA dropped. The best point of balance should be sought between extraction time and temperature for the maximum EYA. Figure 1C shows that EYA reached the maximum value in the middle level of ethanol concentration and temperature. Figure 1D–F indicated liquid-solid ratio possessed a positive effect on EYA. When other factors remain unchanged, the EYA efficiency increased with the elevation of liquid-solid ratio. The reason is that the presence of less liquid prevented the extraction of the whole astilbin. Consequently, the higher EYA efficiency was obtained as a larger volume of water was utilized.

The optimal conditions analyzed using Design Expert 7.0 were shown as an extraction time of 40 min, ethanol concentration of 60%, temperature of 73.63 °C and liquid-solid ratio of 29.89 mL/g with the highest predicted yield of astilbin 15.05 mg/g.

### 2.3. Verification of Predictive Model

In order to validate the adequacy of the model equations, a verification experiment was carried out under the adjusted optimal conditions, which were an extraction time of 40 min, ethanol concentration of 60%, temperature 74 °C and liquid-solid ratio 30 mL/g. The result showed that the extraction yield of astilbin was 14.97 ± 0.04 mg/g, indicating that the extraction conditions obtained by response surface methodology were practical.

### 2.4. Effects of Astilbin on RAW264.7 Cell Viability and Production of Pro-Inflammatory Mediators

During the progress of inflammation, macrophages actively participated in inflammatory responses by releasing the pro-inflammatory cytokines, including TNF-α, IL-6, IL-1β, as well as other inflammatory factors, such as NO, prostaglandin E_2_ (PGE_2_), which recruit additional immune cells to the sites of infection or tissue injury [25,26]. In present study, the anti-inflammatory effect of astilbin was investigated in LPS-induced RAW264.7 macrophages.

**Figure 2:**
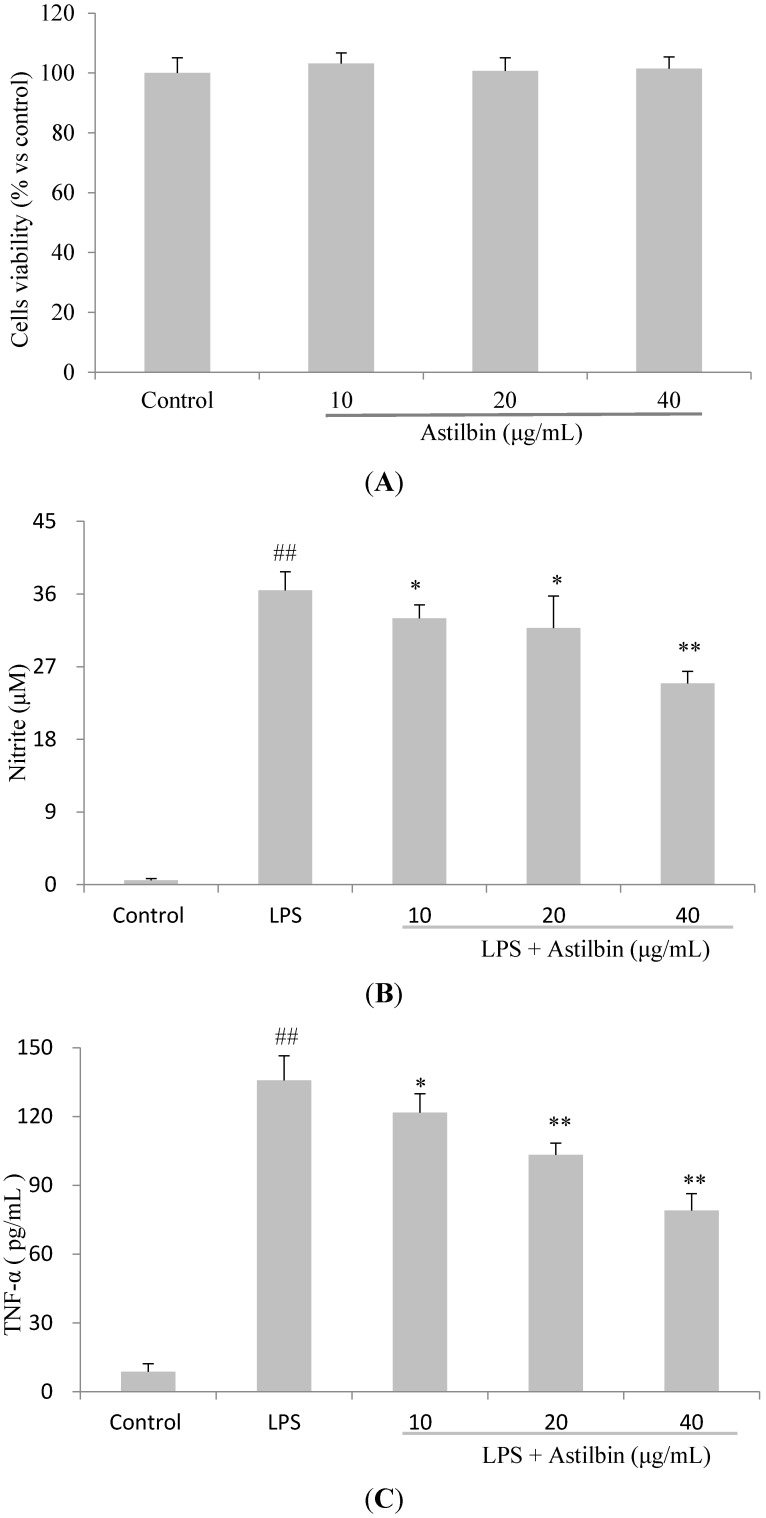

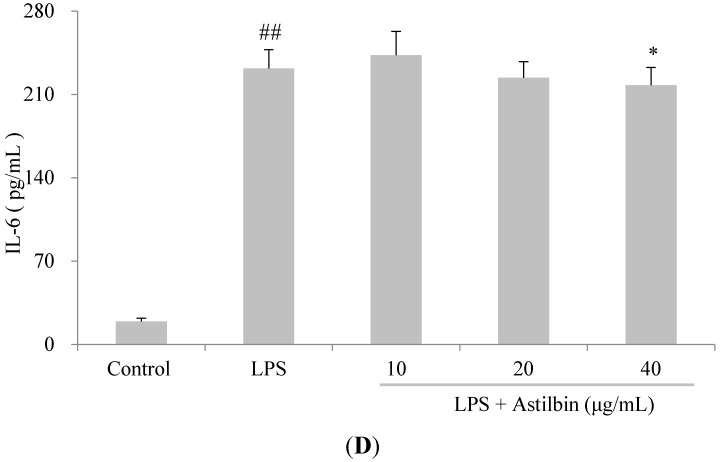
Effects of astilbin on cell viability (**A**) and the production of NO (**B**), TNF-α (**C**), and IL-6 (**D**) in LPS-induced RAW264.7 cells. (A) Cells were treated with different concentrations (10, 20, and 40 µg/mL) of astilbin for 24 h. Cells viability was determined using MTT assay. Results were expressed as mean ± standard deviation (SD) from six independent experiments in each group. (B–D) Cells were incubated with varying concentrations of astilbin for 2 h, and then stimulated with LPS (0.1 µg/mL) for 20 h (for TNF-α and IL-6) or 24 h (for NO), respectively. Culture medium was tested for the production of nitrite, TNF-α and IL-6. The results are expressed as mean ± SD values (n = 3). ## *p* < 0.01 compared to the control group, while * *p* < 0.05 and ** *p* < 0.01 compared to the LPS-treated group.

The viability of RAW264.7 cells in response to astilbin was examined initially for 24 h by 3-(4,5-dimethylthiazol-2-yl)-2,5-diphenyl tetrazolium bromide (MTT) assay. Results indicated that cells viability was not affected by astilbin at concentration of 10–40 µg/mL (Figure 2A). Hence, it was concluded that the inhibition of inflammation was not due to cytotoxicity of astilbin. The effects of different concentrations of astilbin (10, 20, and 40 µg/mL) on LPS-induced NO production in RAW264.7 cells were investigated by measuring the accumulation of nitrite in the culture medium. As expected, LPS stimulation increased the concentration of NO significantly (Figure 2B). Notably, astilbin showed an obvious depression of LPS-induced NO production in a dose-dependent manner (Figure 2B). Consistent with the inhibitory effect on NO production, a dose-dependent suppression of astilbin on TNF-α was also observed (Figure 2C). However, on IL-6 production, astilbin showed a significant inhibitory effect at high concentrations (40 µg/mL), but no obvious effect was seen at lower concentrations (Figure 2D).

**Figure 3:**
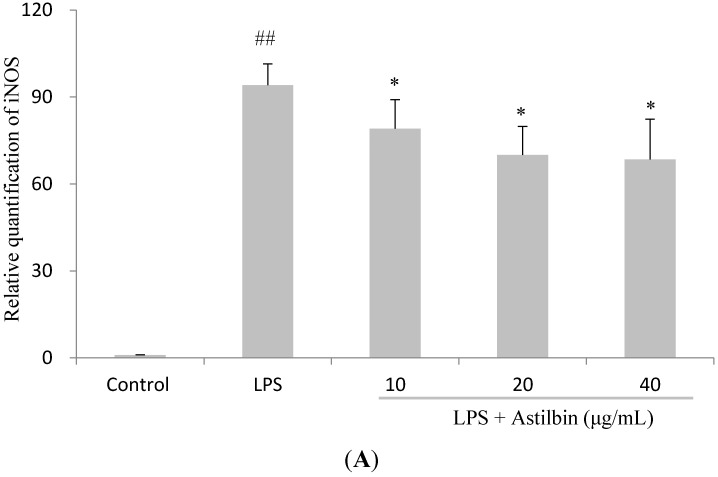

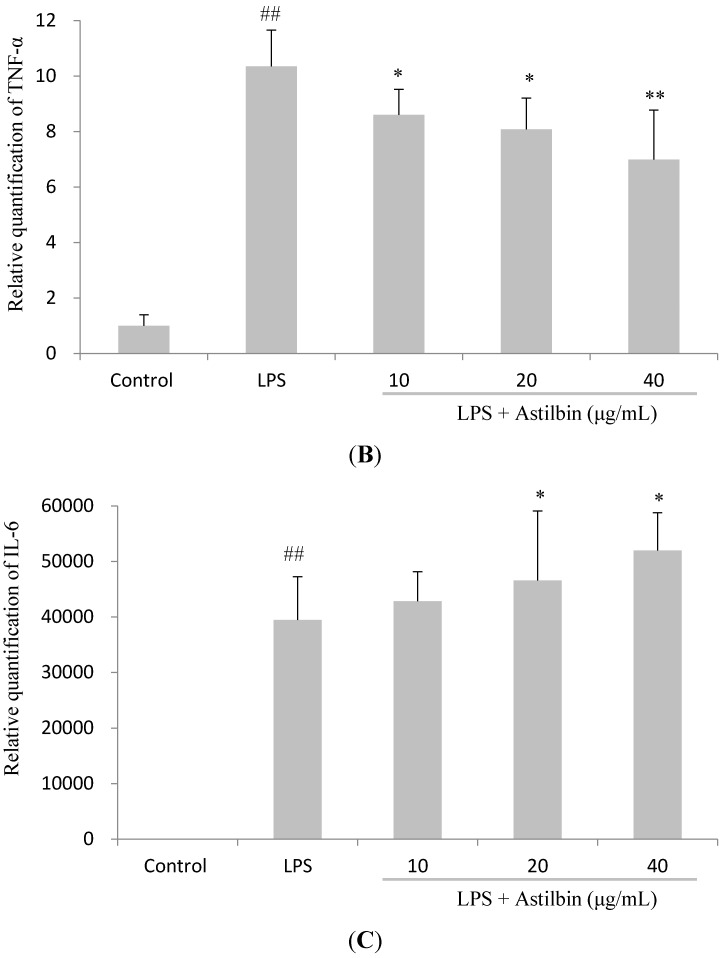
Inhibitory effect of astilbin on iNOS, TNF-α and IL-6 mRNA expressions in LPS-induced RAW264.7 cells. Cells were pretreated with different concentrations of astilbin for 2 h, and then stimulated with LPS (0.1 µg/mL) for 12 h. The relative iNOS (**A**), TNF-α (**B**) and IL-6 (**C**) mRNA expression was performed by real-time RT-PCR, and results were expressed as the ratio of the optimal density relative to glyceraldehydes 3-phosphate dehydrogenase (GAPDH). ## *p* < 0.01 compared to the control group, while * *p* < 0.05 and ** *p* < 0.01 compared to the LPS-treated group.

Since NO produced by macrophages is catalyzed by the iNOS protein, which is reported mainly regulated at the transcriptional level [27]. Thus, the expression level of iNOS was examined by RT-PCR. Results indicated that the expression level of iNOS gene was markedly up-regulated in response to LPS, and astilbin exerted a significant inhibition on this up-regulated expression of iNOS in a dose-dependent manner (Figure 3A). In addition, the effects of astilbin on LPS-induced TNF-α and IL-6 mRNA expression were also measured. The results showed that astilbin displayed a strong effect in inhibiting the LPS-induced TNF-α mRNA expressions in a concentration-dependent manner (Figure 3B). However, on IL-6 mRNA expression, astilbin showed an enhanced up-regulated effect in a dose-dependent manner (Figure 3C). These results suggest that the transcriptional regulation of iNOS and TNF-α gene could be targeted by astilbin.

These results are consistent with the previous report by Huang *et al*., which demonstrated that astilbin isolated from the leaves of *E. roxburghiana* possessed potential inhibitory effect on LPS-induced expression of TNF-α, IL-1β, IL-6, monocyte chemoattractant protein 1 (MCP-1), and cyclooxygenase-2 (COX-2) on transcriptional level in the mouse J774A.1 macrophage cell [9]. In addition, research of Ding *et al.* showed that astilbin could serve as a candidate drug for inflammation disease by mediating the regulatory function of dendritic cells [28]. However, astilbin isolated from active extract of *S. corbularia* exhibited potential inhibitory effect on LPS-induced PGE_2_ release from RAW264.7 cells (IC_50_ = 19.6 µg/mL), but had no effect on NO and TNF-α production (IC_50_ > 100 µg/mL) [29].

### 2.5. Effects of Astilbin on NF-κB and MAPKs Pathways

**Figure 4:**
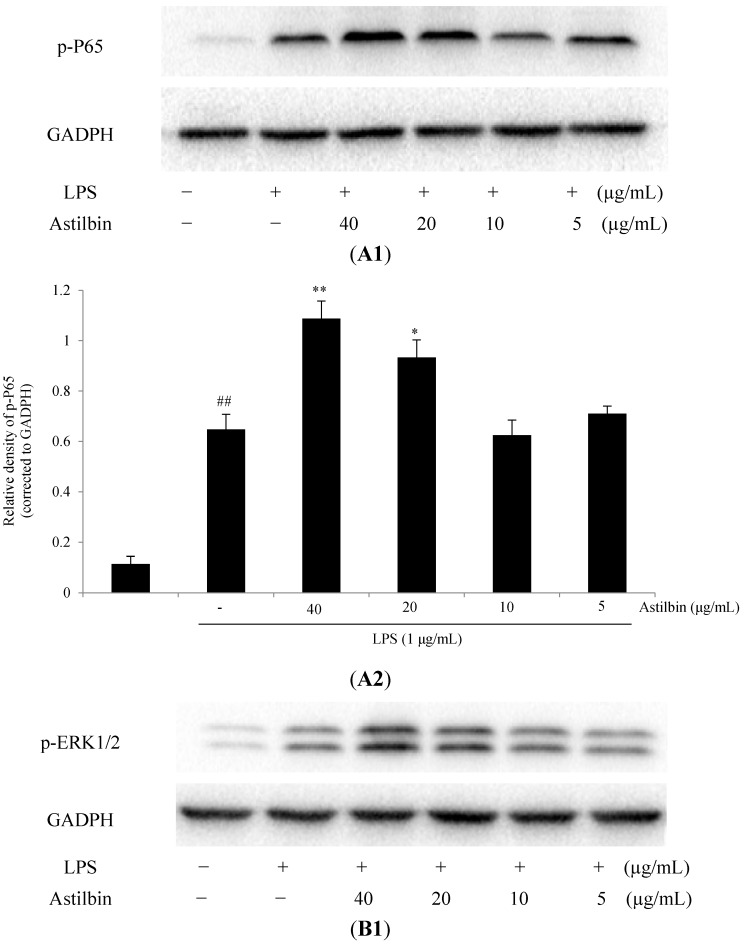

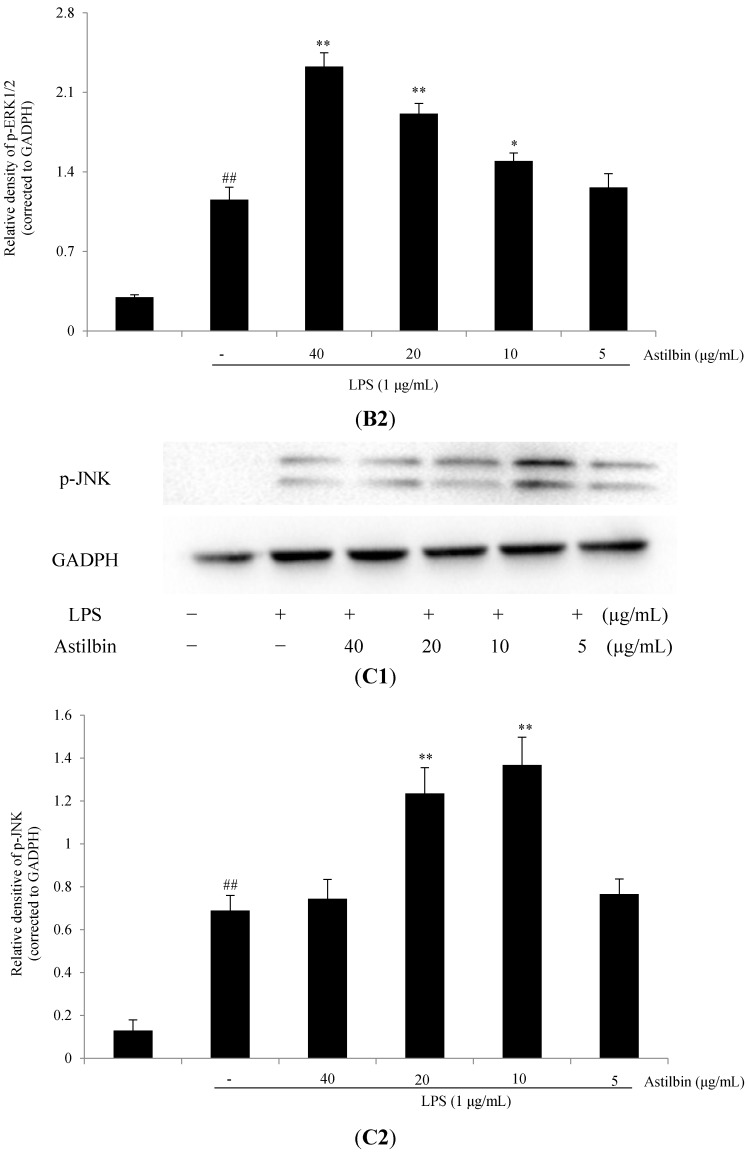

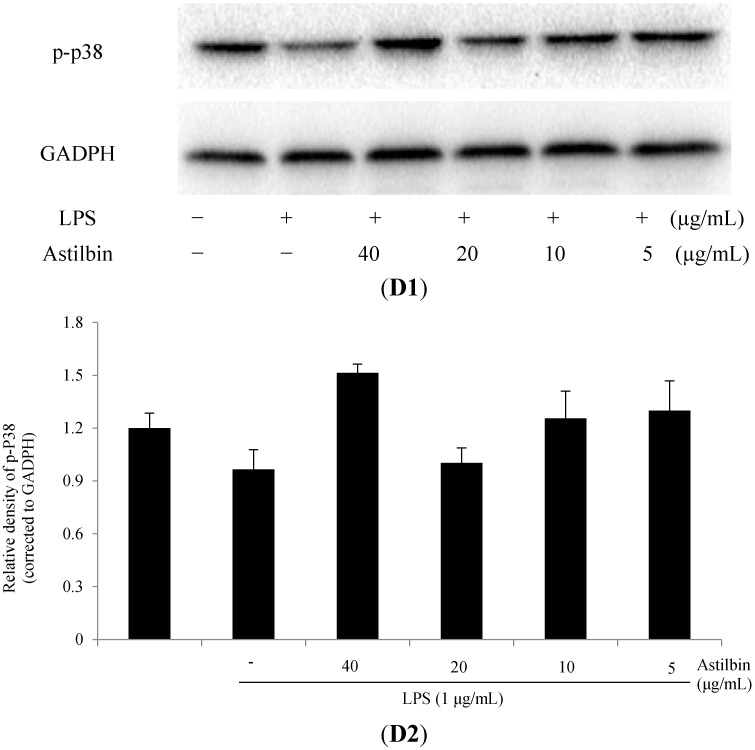
Effects of astilbin on NF-κB and MAPK pathways in LPS-induced RAW264.7 macrophages. Cells were incubated with different concentrations of astilbin (5, 10, 20, and 40 µg/mL) for 1 h before exposure to LPS (final concentration 1 µg/mL) for 1 h. Protein was extracted and analyzed using western blotting. One of three experiments is shown. The protein levels were normalized to the levels of GAPDH. (**A**) Effect of astilbin on the phospho-p65, (**B**) effect of astilbin on the phospho-ERK1/2, (**C**) effect of astilbin on the phospho-JNK, (**D**) effect of astilbin on the phospho-p38 MAPK. The data were represented as the mean ± SD (n = 3). ## *p* < 0.01 compared to the control group, while * *p* < 0.05 and ** *p* < 0.01 compared to LPS-treated group.

In order to determine whether the suppression of inflammatory reactions by astilbin was mediated through NF-κB or MAPKs pathway, the effect of astilbin on the LPS-stimulated phosphorylation of p65, ERK1/2, p38 and JNK in RAW264.7 cells was detected by western blot analysis. Cells were pretreated by astilbin (5, 10, 20, and 40 µg/mL) for 1 h before they were stimulated with LPS (1 µg/mL) for 1 h. Results indicated that the level of phosphorylation of p65 in LPS-induced cells was significantly higher than that of LPS-free group (*p* < 0.01) (Figure 4A1,A2). Astilbin, at high concentrations (20 and 40 µg/mL), exerted an up-regulated effect on phosphorylation of p65, while at low concentration (5 and 10 µg/mL) they showed no obvious effect (Figure 4A1,A2). The similar effects on ERK1/2 (Figure 4B1,B2) and JNK (Figure 4C1,C2) phosphorylation were also observed. However, there was no significant difference of the p38 phosphorylation levels between untreated and LPS-treated groups, as well as between LPS-treated and astilbin+LPS-treated groups (Figure 4D1,D2).

NF-κB and MAPKs were well known for their pivotal role in the inflammatory response. It has been widely recognized that the inhibition of the activities of NF-κB and MAPKs has a positive impact on reducing inflammation response. However, astilbin, in the present study, exerted an enhanced up-regulation for LPS-induced p65, ERK1/2, and JNK phosphorylation levels, but not down-regulation, which was different from the other anti-inflammatory agents. The possible reason of this may be associated with the M1 (classic activation) and M2 (alternative activation) polarization of RAW264.7 macrophages, which represent extremes of a continuum in a universe of activation states. M1 cells are frequently implicated in initiating and sustaining inflammation, while M2 cells are associated with resolution or smoldering chronic inflammation [30,31]. It is well known that LPS could lead to NF-κB activation and production of inflammatory mediators associated with M1 macrophages. However, pre-treatment of astilbin may be, to some extent, able to reverse cells to the M2 polarization, as NF-κB activation also activated a genetic program essential for resolution of inflammation and for M2 polarization [31,32,33]. However, more experiments should be carried out to further reveal the mechanism of its anti-inflammatory activity.

## 3. Materials and Methods

### 3.1. Plant & Materials

The rhizomes of *S. glabra* were purchased from Kangmei Pharmaceutical Co. Ltd. (Guangdong, China) in February 2013 (batch No. 12120527), and were verified by Huang Zhi-hai in the Second Institute of Clinical Medicine, Guangzhou University of Chinese medicine (Guangzhou, China). The samples were air-dried at 45 °C, and then pulverized to pass through 100 mesh sieves to obtain fine powder. Astilbin was purchased from Chengdu Must Bio-technology Co. (Chengdu, China) with the purity more than 98% in high performance liquid chromatography (HPLC) analysis. The RAW264.7 cell line was obtained from the Laboratory Animal Center, Sun Yat-Sen University (Guangzhou, China). Dulbeccos’s Modified Eagle’s Medium (DMEM) and fetal bovine serum (FBS) were produced by Invitrogen-Gibco (Grand Island, NY, USA). MTT, LPS and dimethyl sulfoxide (DMSO) were obtained from Sigma-Aldrich (St. Louis, MO, USA). Mouse TNF-α and IL-6 enzyme-linked immune sorbent assay (ELISA) kits were purchased from R & D Systems, Inc (St. Louis, MO, USA). The anti-phosphorylated NF-κB P65, anti-phosphorylated JNK, anti-phosphorylated ERK1/2, and anti-phosphorylated p38 mouse or rabbit antibodies were purchased from Cell Signaling Technology (Danvers, CO, USA).

### 3.2. Extraction Procedure

About 3 g of pre-prepared powder was weighted and placed into a 200 mL Erlenmeyer flask. Then, it was extracted with selected ethanol concentrations and liquid-solid ratio at a given temperature for a specific extraction time and number of extraction cycles. After filtration through 0.45 mm filter paper, the filtrates were combined and concentrated using a rotary evaporator at 45 °C, and then dissolved by ethanol (60%) to a constant volume (300 mL).

### 3.3. Determination of Astilbin

A chromatography system was used to determine astilbin content in the extraction. RP-C18 column (Alltech, 4.6 mm × 250 mm, 5 µm) was installed in an oven maintained at 30 °C. The mobile phase consisting of 55:45 (v/v) methanol:water (contents 0.2% HCOOH) was degassed with helium and maintained under helium for the duration of the experiments. A flow rate of 1.0 mL·min^−1^ was used and the injection volume was 10 µL. The detector wavelength was at 340 nm. Peak area was used to calculate the quantity of astilbin based on the analysis of the standard curve.

While the concentration of astilbin ranged from 70.6–1130.0 µg/mL, there was a good linear relationship between the concentration of astilbin and its peak area, *Y* = 3.4289*X* + 14.234 (r^2^ = 0.9999), where *Y* is the peak area and *X* is the concentration. On the basis of the astilbin content of the prepared samples measured by the standard curve, EYA (mg·g^−1^) was calculated by the following equation:

(2) $$\text{EYA}\;(\%)=\;\frac{\text{C}\cdot \text{V}}{\text{m}\times 10^{6}}\;\times 100\%$$

where *C* is the concentration of astilbin in the tested sample (µg/mL), V is the volume of sample (mL), and m is the mass of the *S. glabra* (g).

### 3.4. Experimental Design

Based on single factor analysis, a Box-Behnken design (BBD) was employed to identify the relationship between the process independent variables (extraction time, A; ethanol concentration, B; temperature, C; and solid-liquid ratio, D) and the response function (EYA). The experimental range of the selected process variables with their units and notation are given in Table 1. A total of 29 experiments were performed in triplicate according to the BBD matrix in Table 1 and the average values were used in data analysis. The experimental data were analyzed by the software, Design Expert Version 7.0.0 (State-Ease, Minneapolis, MN, USA). The adequacy of the developed model and statistical significance of the regression coefficients were tested using ANOVA. The interaction among the different independent variables and their corresponding effect on the response was studied by analyzing the response surface contour pots.

### 3.5. Determination of the Anti-Inflammatory Efficiency of Astilbin

#### 3.5.1. Cell Culture and Treatment

RAW264.7 cells were maintained in DMEM supplemented with 10% heat-inactivated FBS and 1% penicillin-streptomycin solution in a humidified incubator at 37 °C with 5% CO_2_. The cells were cultured for 2–3 days to reach the logarithmic phase and then used for experiments. The cells were treated with astilbin at different concentrations and then stimulated with LPS for the incubated time. The stock solutions of astilbin were prepared in DMSO, and the final concentration of DMSO was less than 0.1%.

#### 3.5.2. Cell Viability Assay

The cytotoxic effect of astilbin on RAW264.7 cells was evaluated by MTT assay as described previously with a slight modification [34]. Briefly, RAW264.7 cells were seeded in 96-well plate, incubated at 37 °C for 12 h, and given a fresh change of medium. Cells were then treated with or without various concentrations of astilbin 37 °C for 24 h. Then, 20 µL of MTT solution (0.5 mg/mL) was added to the wells and the incubation was continued for another 4 h. The culture medium was removed, and the formazan precipitates were dissolved with 150 µL of DMSO. The optical densities (OD) at 570 nm were measured with a microplate reader.

#### 3.5.3. Measurement of Nitric Oxide Production

RAW264.7 cells (2 × 10^5^ cell/mL) were pre-incubated with different concentrations of astilbin (40, 20, and 10 µg/mL) for 2 h and then exposed to LPS (final concentration 0.1 µg/mL) for 24 h. The nitrite accumulated in culture medium was measured as an indicator of NO production based on the Griess reaction [35]. An aliquot of each supernatant (100 µL) was mixed with the same volume of Griess reagent [0.1% N-(1-naphathyl)-ethylenediamine, and 1% sulfanilamide in 5% phosphoric acid] for 10 min at room temperature. The absorbance of the final product at 525 nm was measured, and the nitrite levels in the samples were calculated from a standard curve with known concentrations of sodium nitrite.

#### 3.5.4. Determination of Production of Pro-Inflammation Cytokines

The amounts of TNF-α and IL-6 in the culture media were determined using ELISA kits according to the method as described in previous studies [36]. Briefly, RAW264.7 cells were cultured in 24-well plates, pretreated for 2 h with different concentrations of astilbin, and then stimulated with LPS for 20 h. The culture supernatants were collected immediately after treatment, spun at 12,000× *g* for 3 min to remove the particulate matter. The levels of TNF-α and IL-6 in each sample were measured according to the manufacturer's instructions.

#### 3.5.5. RNA Extractions and Quantitative Real-Time PCR

RAW264.7 cells (4 × 10^5^ cells/well) were cultured in 6-well plates, incubated at 37 °C for 24 h, and then pretreated with various concentrations of astilbin for 2 h before stimulation with LPS (final concentration 0.1 µg/mL) for 12 h. Total cellular RNA was isolated from cells using TRIzol (Invitrogen, Carlsbad, CA, USA), and stored at −80 °C until use. Quantitative real-time PCR was conducted with the FastStart DNA Master SYBR Green I kit (Roche Diagnostics, Mannheim, Germany). PCR reactions conditions: 4 µL Reaction Buffer (×5), 1 µL Ribolock RNase Inhibitor (20 U/µL), 2 µL (10 mM) dNTPs, 1 µL Revert Aid M-MuLV Reverse Transcriptase (200 U/µL), 1 µL Oligo (dT) 18 primer, 3 µg total RNA, and add water (nuclease free) to total volume 20 µL. PCR conditions: 15 s denaturation at 94 °C, 30 s annealing between 55 and 60 °C, 30 s extension at 72 °C, and a final extension of 10 min at 72 °C. The nucleotide sequences of the primers used were as follows: GAPDH (forward, 5′-TTT GTC CTC ATT TCC TGG TAT G-3′; reverse, 5′-TGG GAT AGG GCC TCT CTT GC-3′); IL-6 (forward, 5′-TAC TCG GCA AAC CTA GTG CG-3′; reverse, 5′-GTG TCC CAA CAT TCA TAT TGT CAG T -3′); TNF-α (forward, 5′-GGG GAT TAT GGC TCA GGG TC-3′; reverse, 5′-CGA GGC TCC AGT GAA TTC GG-3′); iNOS (forward, 5′-CGG CAA ACA TGA CTT CAG GC-3′; reverse, 5′-GCA CAT CAA AGC GGC CAT AG-3′). The results were expressed as the ratio of the optimal density relative to GAPDH.

#### 3.5.6. Western Blot Analysis

The effects of astilbin on NF-κB and MAPKs pathways were detected as reported previously [35,36]. RAW264.7 cells (1 × 10^6^ cells/mL) were cultured in 60 mm culture dish, incubated at 37 °C for 12 h, and then pretreated with various concentrations of astilbin for 2 h before stimulation with LPS (final concentration 0.1 µg/mL) for 2 h. Then cells were collected and washed twice with cold phosphate buffered saline (PBS). Cells were lysed in an ice-cold lysis buffer (50 mM Tris-HCl pH 7.5, 150 mM EDTA, 20 mM NaF, 0.5% NP-40, and 1% TritonX-100) containing a protease inhibitor and phosphatase inhibitor cocktail (Roche Diagnostics, Mannheim, Germany) for 40 min. The homogenates were centrifuged at 12,000 *g* for 10 min at 4 °C, and the supernatant was collected for western blot analysis. Protein concentration in all samples was quantified by the bicinchoninic acid method. Prior to electrophoresis, bromophenol blue and dithiothreitol (DTT, final concentration 10 mM) were added to the sample. About 30 µL of protein from the supernatants was then separated on 10% sodium dodecylsulphate-polyacrylamide gel (SDS-PAGE) and transferred to PVDF membranes. After transfer, the membrane was blocked for 2 h at room temperature with 5% skim milk in TBST buffer (20 mM Tris, 500 mM NaCl, pH 7.5 and 0.1% Tween 20), and then incubated with the primary antibodies (anti-phospho-p38 MAPK, anti-phospho-ERK1/2, anti-phospho-JNK, anti-phospho-p65, and anti-GAPDH) diluted in 1% bovine serum albumin (BSA, Sigma-Aldrich) in TBST for 2 h. the membranes were washed three times with TBST at room temperature and then incubated with an anti-rabbit horseradish peroxidase-conjugated antibody diluted 1:15,000 in TBST for 1 h at room temperature. The membranes were washed three times and the immunoreactive proteins were detected by an enhanced chemiluminescence system (GE Healthcare, Little Chalfont, Buckinghanshire, UK). The results of Western blot analysis were quantified by measuring the relative intensity compared to the control and represented in the relative intensities.

### 3.6. Statistical Analysis

The results are presented as mean ± S.E.M, and statistical analysis was performed using a one-way ANOVA. The difference between the mean values of two groups was assessed by the Student-Newman-Keuls test. The accepted level of significance for the test was *p* < 0.05. All statistical tests were carried out using the SPSS software.

## 4. Conclusions

The optimal ethanol extraction of astilbin from the rhizome of *S. glabra* was identified as: an extraction time of 40 min, ethanol concentration of 60%, temperature of 73.63 °C, and liquid-solid ratio of 29.89 mL/g, with the highest predicted yield of astilbin (15.05 mg/g). In addition, the present research demonstrated the anti-inflammatory potential of astilbin, which significantly inhibited the production of NO and TNF-α, as well as their gene expression levels in LPS-induced RAW 264.7 cells, and the possible underlying mechanism may be associated with p65, ERK1/2 and JNK pathways.
